# Familial Dilated Cardiomyopathy and Sudden Cardiac Arrest: New Association with a *SCN5A* Mutation

**DOI:** 10.3390/genes12121889

**Published:** 2021-11-25

**Authors:** Yolanda Rico, Maria Francisca Ramis, Montse Massot, Laura Torres-Juan, Jaume Pons, Elena Fortuny, Tomas Ripoll-Vera, Rosa González, Vicente Peral, Xavier Rossello, Damià Heine Suñer

**Affiliations:** 1 Cardiology Department, Hospital Universitari Son Espases, 07120 Palma, Spain; mariafrancisca.ramisbarcelo@ssib.es (M.F.R.); jaumea.pons@ssib.es (J.P.); elena.fortuny@ssib.es (E.F.); rosam.gonzalez@ssib.es (R.G.); vicente.peral@ssib.es (V.P.); fjrosello@ssib.es (X.R.); 2 Centre Hospitalier Universitaire de Toulouse, Hôpital de Rangueil, 31400 Toulouse, France; montsemassot@gmail.com; 3 Health Research Institute of the Balearic Islands (IdISBa), 07120 Palma, Spain; laura.torresjuan@ssib.es (L.T.-J.); tripoll@hsll.es (T.R.-V.); damian.heine@ssib.es (D.H.S.); 4 Unit of Molecular Diagnostics and Clinical Genetics, Hospital Universitari Son Espases, 07120 Palma, Spain; 5 Cardiology Department, Hospital Universitari Son Llatzer, 07198 Palma, Spain

**Keywords:** dilated cardiomyopathy, familial dilated cardiomyopathy, genetic study, *SCN5A*, novel mutation

## Abstract

Dilated cardiomyopathy (DCM) has significant morbidity and mortality. Familial transmission is reported in 20–35% of cases, highlighting the role of genetics in this disorder. We present an interesting family in which the index case is a 64-year-old woman who survived a sudden cardiac arrest. She presented left ventricular dilatation and dysfunction, which indicated the presence of DCM, as well as a history of DCM and sudden arrest in her family (mother and sister). Genetic testing identified a heterozygous mutation c.74A > G missense change that causes an amino acid, p.Glu25Gly, change in the N-terminal domain of the *SCN5A* protein. After performing an exhaustive family medical history, we found that this previously not described mutation segregated within the family. All relatives with the DCM phenotype were carriers, whereas none of the noncarriers showed signs of heart disease, so this mutation is the most likely cause of the disease. This is the first time that a variant in the N-terminal domain of *SCN5A* has been associated with DCM.

## 1. Introduction

Dilated cardiomyopathy (DCM) is a myocardial disease characterized by enlargement and impaired contractility of the left or both ventricles in the absence of severe coronary artery disease or abnormal loading conditions, such as valvular heart disease. The estimated prevalence of DCM is 40 cases per 100,000 individuals (1:2500). Acquired causes include myocarditis, alcohol intake, drugs, and toxins, as well as metabolic and endocrine disorders. Genetic factors are an important cause at all ages, and a familial transmission has been reported in 20–35% of cases, most of them with an autosomal dominant inheritance. This entity has significant morbidity and mortality, leading to chronic heart failure and sudden cardiac arrest [1,2].

Many genes have been associated with this disease, mostly encoding structural and sarcomeric myocardial proteins. Most commonly, they involve truncating mutations in titin (*TTN*). Other well-known causal genes of DCM are those encoding lamin A/C (*LMNA*), troponin T2 (*TNNT2*), phospholamban (*PLN*), desmin (*DES*), tropomyosin (*TPM1*), vinculin (*VCL*), and RNA-binding motif protein 20 (*RBM20*) [3,4].

Recently, some genes involved in cardiac electrical excitability, such as *SCN5A*, have also been described as a cause of DCM [5]. The *SCN5A* gene is located on chromosome 3p21 and encodes the cardiac Na+ channel, known as Na_v_ 1.5, a member of the family of voltage-gated ion channels. Mutations in *SCN5A* have been primarily associated with pure arrhythmic disorders, although they can also be associated with structural heart diseases. To date, a few *SCN5A* mutations have been linked to complex arrhythmic disorders and DCM. The *SCN5A* gene is ranked the sixth most common cause of familial DCM, which is characterized by age-dependent penetrance and high prevalence of conduction defects and arrhythmias [5,6,7,8,9,10,11,12].

In this study, we describe the characterization of a novel variant in the *SCN5A* gene, segregating in a Spanish family, as a cause of familial DCM.

## 2. Materials and Methods

A retrospective and observational study was conducted in which patients with a diagnosis of DCM and a finding of the p.Gluc25Gly variant in the *SCN5A* gene were analyzed. From them, family screening was carried out, drawing up a genealogical tree of at least three generations and studying clinically and genetically the first-degree relatives of the affected individuals.

Informed consent was obtained from all patients under the institutional review board policies of the hospital.

Clinical screening was performed with an exhaustive medical history, electrocardiogram (ECG), and transthoracic echocardiogram (TTE) of all family members. Cardiac magnetic resonance imaging (cardiac MRI) with late gadolinium enhancement (LGE) and 24 h Holter monitoring were added in individuals with a clinical phenotype.

CMR was performed using a 1.5 Tesla magnetic resonance (General Electric, Boston, MA, USA). Transthoracic echocardiography was performed using a Vivid E9, Vivid E95 (General Electric, Boston, MA, USA), and Philips IE33 (Philips, Amsterdam, Netherlands) and analyzed with EchoPAC v.113 (General Electric, Boston, MA, USA). LV dimension, mass, systolic function, diastolic function, and valvular disease were assessed in accordance with the recommendations of the European Association of Echocardiography and the American Society of Echocardiography [13].

Genomic deoxyribonucleic acid (DNA) was extracted through peripheral blood leukocytes according to standard protocols. The detected *SCN5A* mutation segregation was studied using Sanger sequencing in the cascade screening of the family. This was performed in the following manner: Primers were designed to amplify exon 2, which contains the variant, and polymerase chain reaction (PCR) was performed using these primers, followed by dideoxy sequencing reaction and analysis using an automatic sequencer (Thermo Fisher, Waltham, MA, USA). The variant in the index case was detected by next-generation sequencing (NGS) of the known coding genome (whole-exome sequence, WES) performed at Centogene (Rostock, Germany). In brief, RNA capture baits were used against approximately 60 Mb of the human exome (targeting >99% of the regions in CCDS, RefSeq, and Gencode) to enrich regions of interest from genomic DNA fragments using the SureSelect kit Agilent Human All Exon V6. The generated library was sequenced on an Illumina platform to a depth of reads of ~100×. Next, the sequence was aligned to the hg19 reference version of the human genome (Genome Reference Consortium GRCh37), first filtering of low-quality reads and possible artifacts, and annotation of variants.

A genetic variant was evaluated based on its frequency (MAF below 1% in gnomAD) and its presence in different databases, such as HGMD^®^ (Cardiff University, Cardiff, UK), ClinVar (NCBI, Bethesda, MD, USA) or CentoMD^®^ (Centogene, Rostock, Germany). Predictive bioinformatics “in silico” tools were also used. A reanalysis of this WES was performed at our laboratory in order to detect additional variants in other genes using the Geneyx software (Geneyx, Tel Aviv, Israel). Variant classification followed the guidelines of the American College of Medical Genetics and Genomics and the Association for Molecular Pathology (ACMG/AMP) [14].

## 3. Results

**Figure 1:**
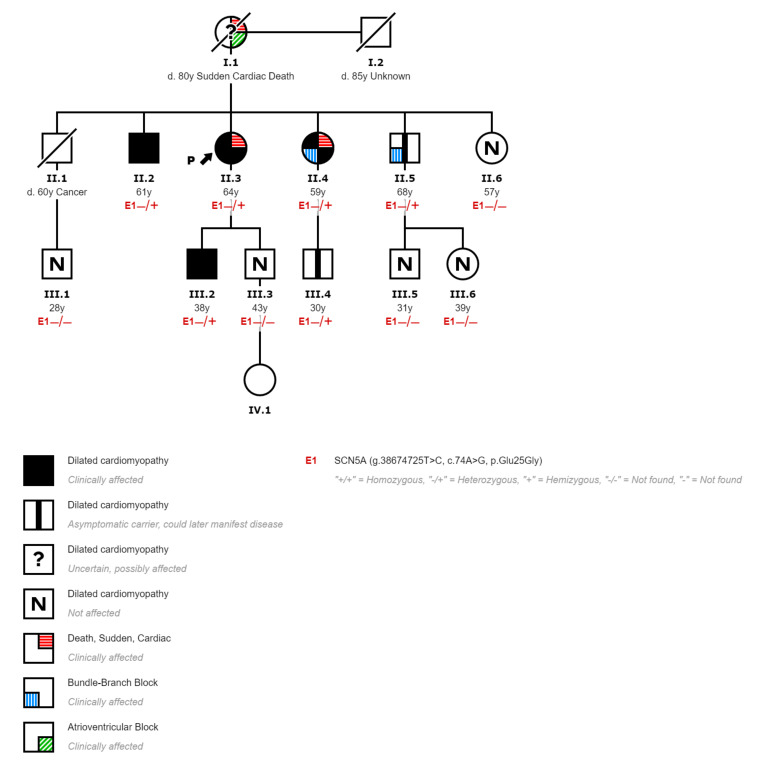
Pedigree of the affected family displaying the segregation of c.74A > G with cardiopathies.

The index case is a 64-year-old woman (II-3, Figure 1) without any known condition who resuscitated from a sudden cardiac arrest secondary to ventricular fibrillation. Her mother, who was using a pacemaker, died suddenly at the age of 80 without an autopsy report (I-1, Figure 1). Her sister also resuscitated from a sudden cardiac arrest due to ventricular fibrillation when she was 59 years old, being diagnosed of DCM then (II-4, Figure 1).

**Figure 2:**
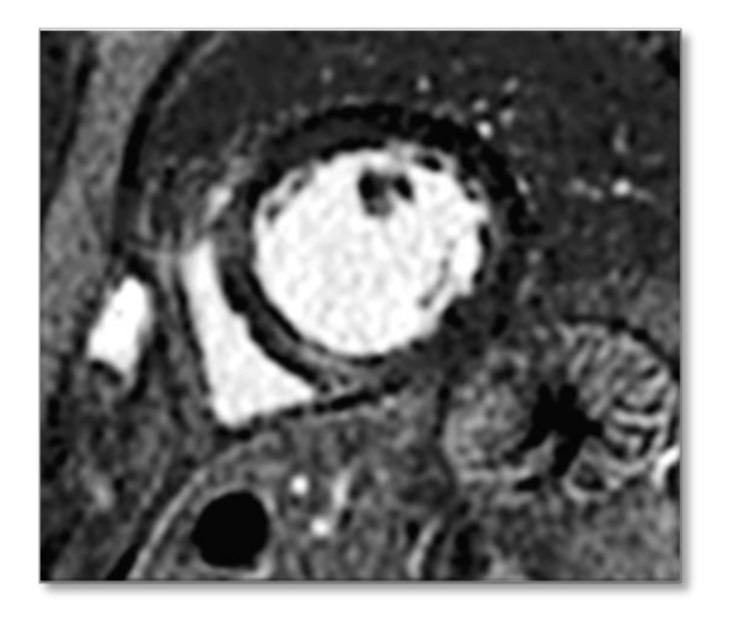
Magnetic resonance image (MRI) of the proband. The arrow shows intramyocardial late gadolinium enhancement (LGE) located in the inferoseptal wall.

A complete clinical history and study of the index case were performed. She worked as a beautician and was able to perform moderate efforts without any cardiovascular symptoms (such as walking for 1 h or going up two flights of stairs without chest pain or breathlessness). The ECG showed a left anterior fascicular block without any other abnormalities. The echocardiogram revealed a severe dilatation of the left ventricle with a severe systolic dysfunction (left ventricular ejection fraction, EF, 28%), a normal right ventricle, and no valvular disease. Coronary arterial disease was ruled out with a coronary angiogram. The study was completed with a cardiac MRI, which confirmed the echo findings and reported the presence of foci of intramyocardial LGE located in the inferoseptal wall representing 14.3% of left ventricular mass (Figure 2). The 24 h ECG monitoring showed multiple episodes of nonsustained monomorphic ventricular tachycardia. The patient was diagnosed of DCM with severe left ventricular dysfunction, and an implantable cardioverter defibrillator was inserted.

The WES of the index case identified a heterozygous NM_198056.2:c.74A>G missense mutation located within exon 2, causing an amino acid substitution, p.Glu25Gly, in the *SCN5A* gene. This is an amino acid conserved position in other species of vertebrates (conservation score, 7.809, from phyloP100way). It has a pathogenic computational verdict based on 12 pathogenic predictions from BayesDel_addAF, CADD, DEOGEN2, EIGEN, FATHMM-MKL, LIST-S2, M-CAP, MVP, MutationAssessor, MutationTaster, Polyphen2-HVAR, and SIFT vs. 1 benign prediction from PrimateAI [15]. Moreover, it has not been detected in either patients or the general population, according to the ClinVar and gnomAD exomes databases. This variant affects an amino acid that is located in the N-terminal domain of the *SCN5A* protein, which has been related to a dominant-negative effect through the interaction of Na_v_ 1.5 alfa-subunits [16], and also seems to be a binding site of calmodulin [17].

A cascade genetic study and phenotypical screening was performed in all living first-degree relatives (4 siblings, 2 sons, 4 niblings). The mutation was found in 3 siblings, 1 son, and 1 nephew. The carrier sister had already been diagnosed of DCM presenting with sudden cardiac arrest due to ventricular fibrillation (II-4, Figure 1). The phenotypical study provided the following features: an ECG with left bundle branch block, a cardiac MRI with mildly dilated left ventricle with moderate systolic dysfunction (EF, 40%) and focal intramyocardial LGE in the inferoseptal wall representing 11% of left ventricular mass, and a 24 h monitorization with multiple nonsustained polymorphic ventricular tachycardia. One carrier brother was diagnosed of DCM with normal ECG, but an echocardiogram that showed a severe dilatation of the left ventricle with normal systolic function (II-2, Figure 1). The carrier son was also diagnosed of DCM with mild dilatation of the left ventricle and mild systolic dysfunction (EF, 46%) (III-2, Figure 1). The other carrier brother had no heart structural abnormalities at the time of the medical screening, although he had an ECG with sinus bradycardia, first-degree atrioventricular block, and right bundle branch block (II-5, Figure 1). One nephew had no heart structural or ECG abnormalities but was also a mutation carrier (III-4, Figure 1). Relatives with no mutation were evaluated with ECG and echocardiogram, and they did not have any pathological phenotype expression (II-6, III-1, III-3, III-5, and III-6, Figure 1).

Therefore, all relatives with a DCM phenotype were carriers of the proband mutation, and none of the noncarriers showed clinical signs of heart disease. Accordingly, it can be assumed that the mutation segregates in the family with an autosomal dominant inheritance, and it is a likely pathogenic mutation according to ACMG/AMP guidelines [14].

## 4. Discussion

This case represents a familial DCM with a novel mutation, p.Glu25Gly, in the *SCN5A* gene. This gene encodes for a subunit of the cardiac sodium channel (Na_v_ 1.5), which is responsible for the initiation and propagation of action potentials and thereby determines cardiac excitability and conduction of electric stimuli through the heart [5,6].

Mutations in this gene have been linked to several diseases. Depending on the type of mutation, and whether it causes gain or loss of function, it can be associated with electrical disorders, such as long QT syndrome, Brugada syndrome, ectopic premature Purkinje-related complexes, isolated cardiac conduction defect, and sick sinus syndrome. However, mutations in *SCN5A* (both gain and loss of function) have also been described in arrhythmogenic cardiomyopathy (almost 2% of cases) and DCM [6,7,8,9].

Since the first cases of DCM associated with *SCN5A* mutations were described, great progress has been made in this area. These mutations are responsible for a modest proportion of DCM (1.7%) and typically present an age-dependent penetrance with phenotype development at an increasing age. Severe heart conduction disturbances and arrhythmias are found in >90% of patients (mainly atrial fibrillation, sick sinus syndrome, premature ventricular complexes, and sometimes ventricular tachycardia) [7,8,9,10,11].

However, the molecular pathways by which *SCN5A* mutations cause ventricular dilatation and dysfunction remain to be elucidated. Several mechanisms have been postulated. Some authors propose that, since Na_v_ 1.5 is a part of a large macromolecular channel complex of regulatory and cytoskeletal proteins, *SCN5A* mutations may disrupt the interactions between Na_v_ 1.5 and other members of this complex, leading to structural deformation and impaired contractility. Other investigators advocate the hypothesis that DCM is secondary to conduction defects because electrical abnormalities usually precede the DCM onset by 15–20 years. Finally, an alternative mechanism could be that DCM results from long-lasting arrhythmias. However, this last mechanism fails to explain why most patients do not exhibit a history of patent arrhythmias [5].

**Figure 3:**
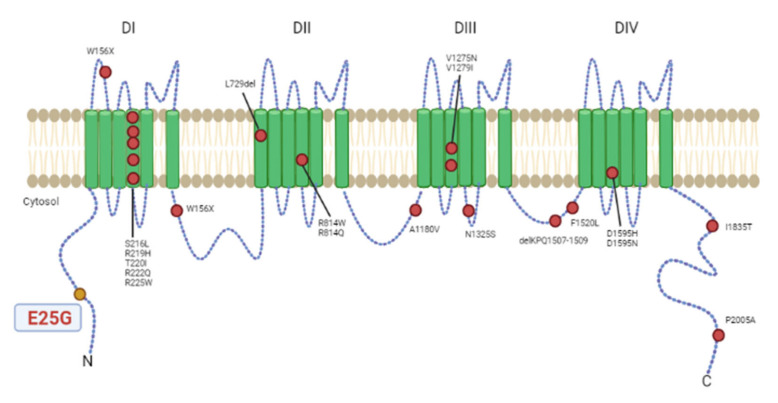
Variants in cardiac Na_v_ 1.5 voltage-gated sodium channel (*SCN5A*) reported previously in association with dilated cardiomyopathy (colored in red). Colored in yellow is the p.Glu25Gly variant described in this article. Two additional variants, c.2550-2551insTG and c.3318dupC, causing truncation of the encoded protein in patients with dilated cardiomyopathy, are not shown. This figure is an adaptation of Asatryan (2019) [12].

Multiple mutations in different positions of the *SCN5A* gene have been described in cases of familial DCM. To our knowledge, this is the first report that describes the p.Glu25Gly mutation in the *SCN5A* gene. Importantly, this mutation seems unequivocally associated with DCM. Only few mutations in *SCN5A* have been described to be associated with DCM, none of them in the N-terminal dominium (see Figure 3). Interestingly, other variants have been described at the N-terminal dominium associated with Brugada syndrome, and it has been shown that some of them exert a dominant negative effect on wild-type (WT) channels since this region is a place of binding to calmodulin [16,17]. The next steps would involve carrying out a functional study of the p.Glu25Gly variant in order to find out whether it produces similar alterations.

The clinical presentation of this familial DCM is typical of *SCN5A* mutations with a marked arrhythmic behavior and a large burden of conduction defects. As discussed above, two of the family members presented with sudden cardiac arrest secondary to ventricular fibrillation and had a conduction disorder; furthermore, another carrier also had a conduction disorder. On the other hand, the deceased mother (I-1, Figure 1) had also been treated with a pacemaker, and although we do not have genetic or phenotypic information and since the father (I-2, Figure 1) did not have any cardiovascular disease, she must be an obligate carrier of the mutation.

Another typical feature of familial DCM with *SCN5A* mutation that occurs in the family described is age-dependent penetrance. The onset of symptoms (sudden cardiac arrest) was at age older than 50 years in the two relatives who had presented it. Furthermore, the youngest carrier of the mutation (30 years old) is the only one with a negative phenotype with a normal TTE and no conduction disorders. This age-dependent penetrance is important to decide the follow-up of the asymptomatic carriers due to the aggressive form of the disease.

In summary, we described a novel variant in the *SCN5A* gene that alters an amino acid in the N-terminal domain in a family with a history of arrhythmia and DCM. This is the first time that a variant in the N-terminal domain of *SCN5A* has been associated with DCM.

## Data Availability

The data that support the findings of this study are available from the corresponding author upon reasonable request.
